# Utilizing Anaerobic Fungi for Two-stage Sugar Extraction and Biofuel Production from Lignocellulosic Biomass

**DOI:** 10.3389/fmicb.2017.00635

**Published:** 2017-04-11

**Authors:** Abhaya Ranganathan, Olivia P. Smith, Noha H. Youssef, Christopher G. Struchtemeyer, Hasan K. Atiyeh, Mostafa S. Elshahed

**Affiliations:** ^1^Department of Microbiology and Molecular Genetics, Oklahoma State University, StillwaterOK, USA; ^2^Department of Biosystems and Agricultural Engineering, Oklahoma State University, StillwaterOK, USA

**Keywords:** anaerobic gut fungi, lignocellulosic biomass, second generation biofuels

## Abstract

Lignocellulosic biomass is a vast and underutilized resource for the production of sugars and biofuels. However, the structural complexity of lignocellulosic biomass and the need for multiple pretreatment and enzymatic steps for sugar release renders this process economically challenging. Here, we report a novel approach for direct, single container, exogenous enzyme-free conversion of lignocellulosic biomass to sugars and biofuels using the anaerobic fungal isolate strain C1A. This approach utilizes simple physiological manipulations for timely inhibition and uncoupling of saccharolytic and fermentative capabilities of strain C1A, leading to the accumulation of sugar monomers (glucose and xylose) in the culture medium. The produced sugars, in addition to fungal hyphal lysate, are subsequently converted by *Escherichia coli* strain K011 to ethanol. Using this approach, we successfully recovered 17.0% (w/w) of alkali-pretreated corn stover (20.0% of its glucan and xylan content) as sugar monomers in the culture media. More importantly, 14.1% of pretreated corn stover (17.1% of glucan and xylan content) was recovered as ethanol at a final concentration of 28.16 mM after the addition of the ethanologenic strain K011. The high ethanol yield obtained is due to its accumulation as a minor fermentation end product by strain C1A during its initial growth phase, the complete conversion of sugars to ethanol by strain K011, and the possible conversion of unspecified substrates in the hyphal lysate of strain C1A to ethanol by strain K011. This study presents a novel, versatile, and exogenous enzyme-free strategy that utilizes a relatively unexplored group of organisms (anaerobic fungi) for direct biofuel production from lignocellulosic biomass.

## Introduction

Production of biofuels from lignocellulosic biomass is regarded as an indispensable component of future sustainable energy landscape scenarios. Lignocellulosic biomass represents a vastly underutilized source for biofuels production, given its availability, low cost, and high-energy content. Nevertheless, lignocellulosic biofuels currently represents an extremely minor component of overall, renewable, or even biofuel-based energy output (Dale, 2015).

The most common approach for biological biofuels production from lignocellulosic biomass utilizes a suite of purified enzymes to release monomeric sugars (mostly glucose and xylose) from pretreated biomass, with the produced sugars converted to biofuels by a dedicated sugar-metabolizer. Various aspects of this strategy have been extensively investigated, benefiting from generous public, private, and public–private partnership funding mechanisms. While significant advances have been achieved in plant genetic engineering (Furtado et al., 2014), pretreatment procedure (Alizadeh et al., 2005; Dadi et al., 2006; Balan et al., 2009), and enzymes discovery and characterization (Hess et al., 2011; Brunecky et al., 2013) remains significantly high (National Research Council, 2011). For example, a recent study estimates theoretical costs of $2.36–2.71 per gallon ethanol under best case scenarios (Johnson, 2016). This is mainly due to the high cost of cellulases, hemicellulases, and accessory enzymes required for the degradation of structurally complex substrates, the high cost and/or operational complexity of pretreatment approaches required to improve enzymes access to lignocellulosic biomass (Alizadeh et al., 2005; Dida et al., 2006; Balan et al., 2009; George et al., 2014), and the operational complexity of the process necessitated by differences in optimal temperatures and/or redox requirements at various stages of the process and frequent formation of inhibitory products during biomass pretreatment (Alvira et al., 2010).

The utilization of microorganism(s) in-lieu of purified enzyme cocktails for breakdown of lignocellulosic biomass represents a promising alternative strategy, since it potentially alleviates many of the problems associated with exogenous enzymes-based procedures. Significant savings could be achieved by eliminating enzymes costs, avoiding harsh plant biomass pretreatments, and process consolidation (Olson et al., 2012). Efforts on this front are geared either toward utilization of a single microorganism, e.g., *Clostridium thermocellum, Clostridium phytofermentans*, and *Caldicellulosiruptor bescii* for complete saccharification and fermentation of pretreated biomass (Jin et al., 2012; Chung et al., 2015), or toward the design of microbial consortia, e.g., *Trichoderma reesei* and *Escherichia coli* for simultaneous saccharification and fermentation of plant biomass by distinct members of the consortium (Minty et al., 2013; Brethauer and Studer, 2014).

Undoubtedly, the success of such strategy necessitates the identification and utilization of robust and invasive biomass degrading-anaerobes, as well as the design of a robust and stable platform for optimal allocation of lignocellulosic substrate utilized between microbial growth, extracellular enzymes production, and desired end products. Members of the anaerobic gut fungi (Phylum Neocallimastigomycota) are one of the most efficient, yet-largely overlooked, anaerobic biomass degraders (Youssef et al., 2013; Gruninger et al., 2014). Anaerobic gut fungi reside in the rumen, hindgut, and feces of ruminant and non-ruminant herbivorous mammals and reptilian herbivores, where they produce a wide array of cell-bound and cell-free cellulolytic, hemicellulolytic, glycolytic, and proteolytic enzymes (Ljungdahl, 2008). Axenic cultures of anaerobic fungi have been shown to metabolize a significant fraction of plant biomass substrates in minimal media (Youssef et al., 2013; Liggenstoffer et al., 2014). Sugars generated during biomass saccharification by anaerobic fungi are metabolized using mixed acid fermentation reaction where lactate, formate, acetate, and hydrogen are produced as major fermentation end products. In addition, a minor amount of ethanol ranging between 0.02 and 0.1 g/g substrate metabolized is typically produced cytosolically from acetyl-CoA using an aldehyde dehydrogenase/alcohol dehydrogenase enzyme system (Boxma, 2004; Youssef et al., 2013).

The efficient biomass-degradation capabilities of anaerobic fungi render them promising agents for biofuel production from lignocellulosic biomass. However, the predominance of acids rather than alcohols as fermentation end products precludes their utilization in axenic monocultures. Here, we explore the utility of an anaerobic gut fungal isolate (*Pecoramyces ruminantium* strain C1A, henceforth referred to as C1A) for direct production of sugars and biofuels from lignocellulosic biomass.

## Materials and Methods

### Microorganisms

*Pecoramyces ruminantium* strain C1A (Hanafy et al., unpublished) was isolated from the feces of an Angus steer and maintained by continuous subculturing into anaerobic fungal media as previously described (Youssef et al., 2013). Strain K011 was purchased from the American Type Culture Collection (ATCC5214^TM^) and maintained on LB Agar with 2% glucose and 40 mg/l chloramphenicol. Strain K011 is a publicly available genetically engineered strain that stoichiometrically converts glucose or xylose to ethanol and two CO_2_ molecules (Ingram et al., 1987).

### Plant Materials and Pretreatment

Corn stover (*Zea mays*) was obtained from Industrial Agricultural Products Centre at University of Nebraska, Lincoln, NB, USA. Mature Kanlow Switchgrass (*Panicum virgatum* var. *Kanlow*) was obtained from Oklahoma State University experimental plots, Stillwater, OK, USA. Mature Sorghum forage (*Sorghum bicolor)* and mature energy cane (*Saccharum officinarum* var. Ho02) was obtained from Oklahoma State University experimental plots in Stillwater, OK, USA. Samples of virgin biomass (mixed tallgrass prairie native to the Great Plains) were a mixture of a C3 grass (Canada wildrye, *Elymus Canadensis* L), a C4 grass (Tall dropseed, *Sporobolus compositus*) and a forb (Western ragweed, *Ambrosia psilostachya*) species. These samples were collected from the West John Lee site at the Tallgrass Prairie Preserve (38.43° N, 96.56° W, Osage County, OK, USA) in August 2013. This particular patch was last burnt in spring 2011. Samples were dried overnight at 45°C, milled, sieved to a particle size of 2 mm (0.5 mm for mixed prairie grasses), as previously described (Suryawati et al., 2008) prior to pretreatment. Alkaline pretreatment was conducted by incubating plant biomass (10% w/v) at 121°C for 1 h with 40 ml of 3% NaOH solution. The pretreated plant biomass was then washed with two liters of deionized water to remove excess alkali and water-soluble components. Treated biomass varied between 68.8 and 70.8% of the dry weight of the original biomass material. All pretreated biomass were dried at 45°C for approximately 48 h before usage in subsequent experiments.

### Process Overview and Experimental Set-up

**FIGURE 1:**
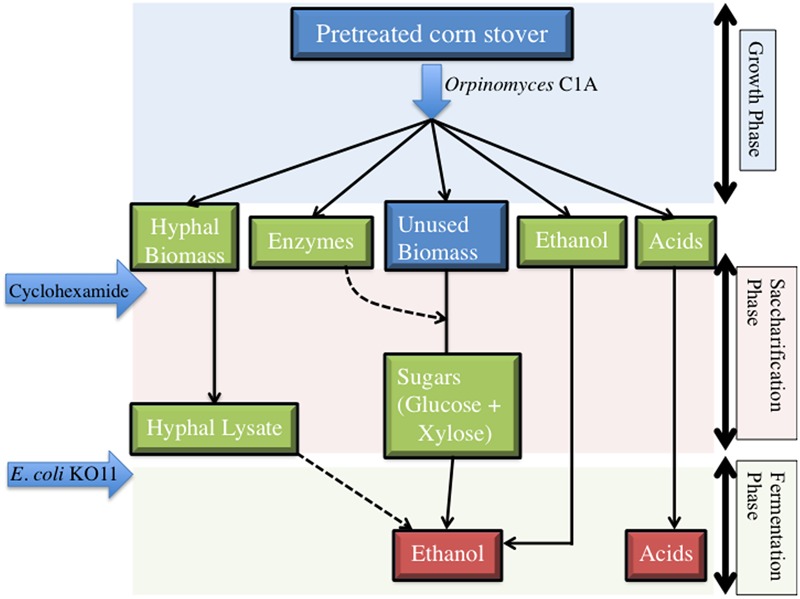
**Overview of the process utilized for sugar extraction and biofuel production from lignocellulosic biomass**.

The utilized approach is shown in **Figure 1** and involves three phases: (1) *Growth phase:* Strain C1A is allowed to grow on lignocellulosic biomass. During this initial phase, strain C1A produces hyphal biomass that effectively colonizes and penetrates the cell walls of plant substrates. More importantly, C1A growth is associated with the production of extracellular lignocellulolytic enzymes that attack the cellulose and hemicellulose fraction of plant biomass as previously demonstrated (Youssef et al., 2013; Couger et al., 2015). Minor amounts of ethanol, in addition to volatile fatty acids (formate, lactate, and acetate) are produced as end products of C1A fermentation during this phase. (2) *Saccharification phase:* Growth of strain C1A is arrested using either atmospheric air exposure or cycloheximide addition, as described below. However, the activity of the stable, extracellular, and oxygen-indifferent plant biomass degradation enzymes is not affected, leading to the gradual accumulation of glucose and xylose in growth media. (3) *Fermentation phase:* Sugars accumulating during the saccharification phase are then metabolized to ethanol using strain K011. Ethanol produced at the conclusion of this scheme is hence generated from K011 metabolism of sugars (and putatively selected compounds in C1A hyphal lysate), as well as from C1A metabolism during the initial growth phase.

### Experimental Set-up

Experiments were conducted in a defined, rumen-free medium (45 ml) in 160 ml serum bottle (Youssef et al., 2013). All media were prepared under strict anaerobic techniques, under a headspace of 100% CO_2_ and were amended with L-cysteine hydrochloride (0.05 g/l final concentration) as a reductant, and resazurin (0.0001% final concentration) as a redox indicator. The media was autoclaved, cooled, then transferred to an anaerobic chamber (Coy Laboratory Products Inc., Ann Arbor, MI, USA), where pretreated biomass (≈ 0.5 g) was added. The media was taken out of the chamber and the headspace of the bottles was changed by repeated flushing with 100% CO_2_. Subsequently, 5 ml (approximately 2.6 mg) of actively growing culture of C1A was used as inoculum. The cultures were incubated at 39°C under stationary conditions.

To terminate the growth phase and initiate the saccharification phase, growth of strain C1A was arrested. Optimal time for growth arrest was empirically determined. Two inhibition methods were evaluated: Aeration, and the addition of a general fungal inhibitor (cycloheximide, 1 mg/ml final concentration). Both approaches arrest C1A growth, sugar uptake and central metabolism, and induce hyphal lysis while not impacting the activities of extracellular poly-saccharolytic enzymes.

At the conclusion of the saccharification phase (**Figure 1**), strain K011 was added to initiate the fermentation of produced sugars. Strain K011 cells were grown overnight (16 h) in LB media till the late log phase, washed three times in PBS buffer, and inoculated to a final concentration of 4 × 10^8^ cells/ml.

### Analytical Methods

Glucose, xylose, fatty acids (acetate, lactate, and formate) and ethanol content in the liquid fractions of the microcosms were quantified by HPLC (Agilent 1100 series, Santa Clara, CA, USA) fitted with a refractive index detector (RID). Monomeric sugar samples were run through an Aminex HPX-87P column (Bio-Rad, Sunnyvale, CA, USA), set at 80°C, using deionized water at a flow rate of 0.6 ml/min as the mobile phase with a 30 min run time as previously described (Liggenstoffer et al., 2014). In addition, glucose and ethanol were also quantified using the PGO (glucose oxidase/peroxidase) (Sigma–Aldrich, St. Louis, MO, USA), and the EnzyChrom (Bioassay Systems, Hayward, CA, USA) kits, respectively, as per the manufacturers’ instructions. Fatty acid (acetate, lactate, and formate) and ethanol were quantified using an Aminex HPX-87H (Bio-Rad, Sunnyvale, CA, USA) column. The column was set at 60°C, with 0.01 N H_2_SO_4_ as the mobile phase with a flow rate of 0.6 ml/min. Compositional analysis of pretreated substrates was conducted as described previously (Youssef et al., 2013). Fungal biomass was quantified indirectly by measuring headspace gas pressure and also by measuring amount of formate, and correlating these fermentation products to the produced fungal biomass using a standard curve constructed on a soluble substrate (cellobiose) as described previously (Lowe et al., 1987; Theodorou et al., 1994). To quantify the total fungal protein concentration, we separated the cell pellet from the cell-free supernatant using centrifugation. The cell pellet fraction was lysed by crushing in a sterile mortar upon submersion in liquid nitrogen, and the lysate was used for total protein extraction using tris-glycine extraction buffer (g/L: Tris base, 3 g; glycine, 14.4 g, pH 8.3). The cell-free supernatant was used directly for protein quantification. Total protein in both fractions was quantified using the Qubit^®^ protein assay kit (Life technologies^®^, Carlsbad, CA, USA).

### Enzymatic Assays

Endoglucanase, exoglucanase, and xylanase activities were determined using a DNS (3,5-dinitrosalicyclic acid)-based assay (Breuil and Saddler, 1985), with carboxymethyl cellulose sodium salt (CMC, 1.25% w/v), avicel microcrystalline cellulose (1.25% w/v), and beechwood xylan (1.25% w/v) as substrates, respectively. Assays were conducted for 2 h in a sodium acetate buffer (100 mM). Cellobiohydrolase, β-xylosidase, and α-*N*-arabinofuranosidase activities were determined using (10 mM) of the *p*-nitrophenol-based (PNP) substrates: *p*-nitrophenyl-β-D-cellobioside (PNPC,), *p*-nitrophenyl-β-D-xylopyranoside (PNPX), *p*-nitrophenyl-β-D-arabinofuranoside (PNPA), respectively (Kubicek, 1982; Dashtban et al., 2010). Assays were conducted for 15 min in sodium acetate buffer (50 mM), with sodium carbonate (1M) as a stop reagent. β-Glucosidase activity was assayed using the β-glucosidase Activity Assay Kit (Sigma–Aldrich, St. Louis, MO, USA), and α-glucuronidase activity was assayed using the Megazyme α-glucuronidase assay kit (Wicklow, Ireland) per manufacturers’ instructions. All enzyme activities were reported as U/mg protein, where a 1U is defined as the number of μmoles of product released per minute.

## Results

Preliminary experiments evaluating C1A growth on alkali-treated corn stover was conducted to determine the optimal time for arresting growth and initiating saccharification (**Figure 1**). Inhibition after 48 h of C1A growth yielded the highest free sugars (glucose + xylose) per gram pretreated corn stover (Supplementary Table S1). The method of fungal growth inhibition (air exposure or cycloheximide addition) had no clear effect on the sugar/corn stover ratio (Supplementary Table S2). Cycloheximide addition was chosen as the preferred method of inhibition since aeration could lead to introduction of airborne contaminants as well as partial loss of ethanol produced during the initial C1A growth phase.

**FIGURE 2:**
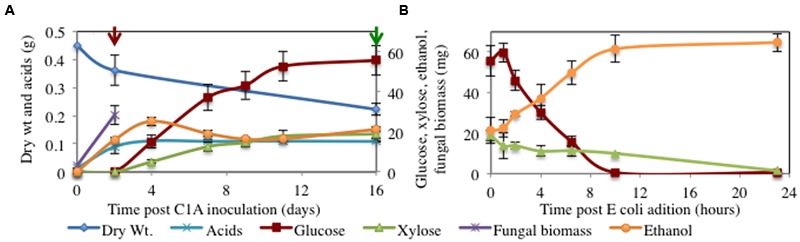
**(A)** C1A growth on alkaline pretreated corn stover. During the initial growth phase (Day 0–2), strain C1A is grown under anaerobic conditions on corn stover, a process that is accompanied by the production of acids and ethanol. Cycloheximide is added after 2 days (red arrow) to initiate the saccharification phase, a process that results in the accumulation of glucose (at a rate of 308 ± 54 μg.h^-1^) and xylose (at a rate of 82.6 ± 11.9 μg.h^-1^) in the culture media. Green arrow depicts the time of addition of *E. coli* to initiate the fermentation phase of the process. **(B)** Addition of *E. coli* results in the rapid consumption of glucose and xylose in the culture media, and their conversion to ethanol (at a rate of 3907 ± 674.9 μg.h^-1^).

**FIGURE 3:**
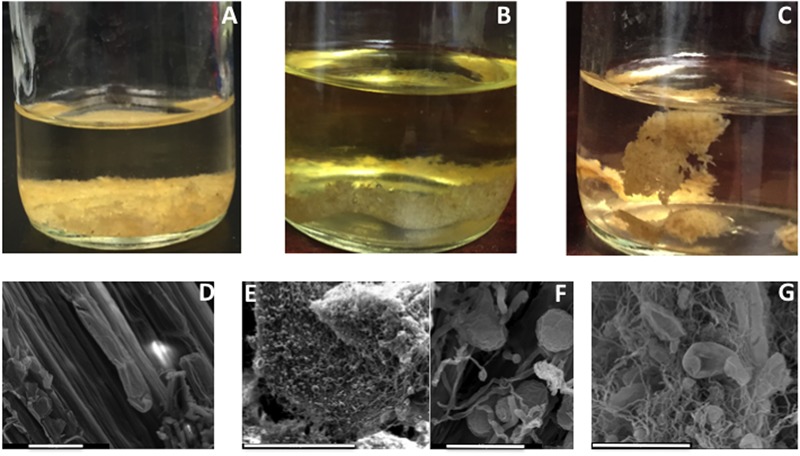
**Culture media with pretreated alkaline corn stover (A)**, with scanning electron micrograph (SEM) showing the intact structure of its particles prior to fungal inoculation (**D**, scale bar 50 μM). Growth of strain C1A on alkaline-pretreated corn stover for 2 days resulted in visual growth around corn stover particles **(B)**, with extensive sporangia and rhizoidal colonization (**E**, scale bar 300 μM) that appears closely associated and penetrating corn stover particles (**F**, scale bar 30 μM). At the conclusion of the saccharification phase, the loss of corn stover weight and density could be visually ascertained **(C)**, with SEM (**G**, scale bar 50 μM) showing sporangial and rhizoidal remains, as well as pronounced pitted patterns (arrow) suggesting extensive decay of corn stover particles.

We subsequently evaluated the ability of strain C1A to extract glucose and xylose from 450 mg of alkali-pretreated corn stover (**Figure 2A** and **Table 1**). During the growth phase (days 0–2), production of hyphal biomass (28.3 mg, **Figures 3A–D**) and free non-adsorbed extracellular proteins (8.9 mg total protein measured in the cell-free supernatant) was observed, as well as the accumulation of C1A fermentation end products, namely acids (79.53 mg acetate, 5.76 mg lactate, and 4.14 mg formate), and ethanol (21.29 mg, 9.2 mM). Upon inhibition of growth after 48 h, fungal biomass and acids/ethanol production ceased (**Figure 2A**), and lysis of fungal hyphae was observed (**Figures 3D–G**). Inhibition of C1A growth also resulted in arresting sugar uptake and fermentation by C1A, but not the plant biomass saccharification process, mediated by extracellular and cellulosomal enzymes produced during C1A growth phase. The saccharification phase was thus associated with the gradual release of glucose (55.7 mg, at a rate of 6.16 ± 1.1 μg (ml h)^-1^ and xylose (18 mg, at a rate of 1.65 ± 0.24 μg (ml h)^-1^ into the culture media. No gluco- or xylo-dimers or oligomers were identified in the culture media. At the conclusion of the growth and saccharification phases, 74.6 mg sugars, and 21.29 mg ethanol accumulated in the culture media (**Figure 2A**). The sugars released represent 17% of starting NaOH-pretreated corn stover dry weight and 20.0% of the glucan and xylan content of corn stover, while the released ethanol represents 4.73% of corn stover dry weight and 5.7% of glucan and xylan content of corn stover (**Table 1**).

**Table 1:** Sugar release from alkaline pretreated corn stover by strain C1A.

Corn stover dry weight (mg)			Products (mg)^2^							Recovery percentages^3^					
T_0_	T_f_^1^	% Lost	Acids^4^	Ethanol	Fungal biomass	Glucose	Xylose	Total sugars	Extracellular proteins^5^	g sugar/ g corn stover	g sugar/ g corn stover	Glucose yield^6^	Xylose yield^7^	Ferment-able sugars yield^8^	Sugars: acids ratio^9^
450	220	51.11	89.42 ± 23.7	21.29 ± 6.56	28.29 ± 4.6	55.66 ± 7.42	18.93 ± 2.52	74.59	8.9	0.17	0.324	22.36	12.36	19.98	0.83

*^1^Dry weight measured at the conclusion of saccharification phase. ^2^All products are measured at the conclusion of saccharification phase, with the exception of fungal biomass, which was measured at the end of the growth phase, since hyphal lysis occurred during saccharification. ^3^Recovery percentages are based on glucan, xylan, and lignin content determined for various lignocellulosic biomass utilized in this experiment and reported in Youssef et al. (2013). ^4^Values represent cumulative amounts of acetate (79.53 ± 23.53), lactate (5.76 ± 0.07), and formate (4.14 ± 0.06) produced. ^5^Value corresponds to the free non-adsorbed extracellular proteins measured in the cell-free supernatant. ^6^Calculated using the formula: [(g glucose produced/g glucan in starting corn stover)] × 100. ^7^Calculated using the formula: [(g xylose produced/g xylan in starting corn stover)] × 100. ^8^Calculated using the formula: [g glucose + xylose produced/g cellulose and glucan in starting corn stover] × 100. ^9^Calculated using the formula: [g glucose + xylose produced/g acetate + formate + lactate produced] × 100.*

**FIGURE 4:**
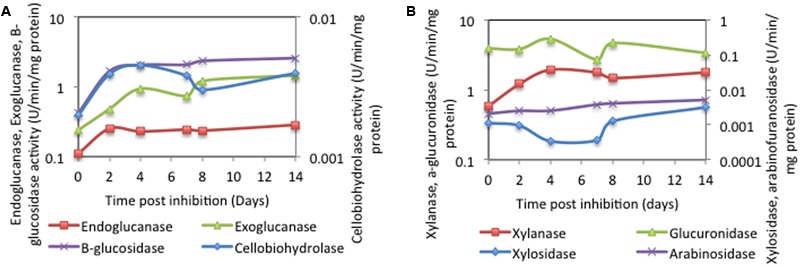
**Stability of (A)** cellulolytic and **(B)** xylanolytic C1A enzymes post C1A growth inhibition by cycloheximide. Values are reported in U/mg proteins.

We monitored the production and stability of eight different enzymatic activities (endo-β-1,4-glucanase, exo-β-1, 4-glucanases, cellobiohydrolase, and β-glucosidase for cellulose degradation, and endo-β-1,4-xylanase, xylosidase, α-glucuronidases, and α-*N*-arabinofuranosidase for arabi-noxylan degradation) during the saccharification phase in the liquid as well as the pellet (plant plus associated hyphal biomass fractions). All eight different activities were identified in both fractions (**Figure 4** and Supplementary Tables S3, S4), with the exception of α-*N*-arabinofuranosidase, identified only in the pellet fraction. More importantly, all measured activities were stable throughout the saccharification phase, strongly indicating the stability of C1A lignocellulosic enzymes under examined conditions.

We sought to determine whether the released sugars could be metabolized within the same container by the addition of a sugar metabolizer. To this end, we tested the ability of *E. coli* strain K011 to convert the produced sugars into ethanol. K011 addition resulted in the rapid (10 h for glucose, 23 h for xylose) and complete conversion of both sugars into ethanol, with glucose fermentation preceding xylose (**Figure 2B**). Remarkably, the ethanol produced in the fermentation phase (43.49 mg, at a rate of 78.14 ± 13.50 μg⋅(ml⋅h)^-1^, 0.58 g/g sugar) exceeds the theoretical values (0.51 g/g sugar) obtained through glycolysis and subsequent production of 2 moles ethanol/mole sugar. We attribute this increase to the fact that cellular components released during C1A hyphal lysis during the saccharification phase could contribute to strain K011 growth and product formation (**Figure 2B**). Further, it is possible that a fraction of the glucose and xylose produced during saccharification is adsorbed to the surface of hyphal biomass, resulting in an underestimation of the proportion of sugars released during saccharification. At the conclusion of the fermentation phase, 64.78 mg ethanol (21.29 mg during growth phase and 43.49 mg during fermentation phase, 28.16 mM final concentration) accumulated in the culture media, corresponding to 14.1% of the dry weight and 17.1% of the total fermentable substrates (glucan and xylan) in corn stover (**Figure 2B**).

**FIGURE 5:**
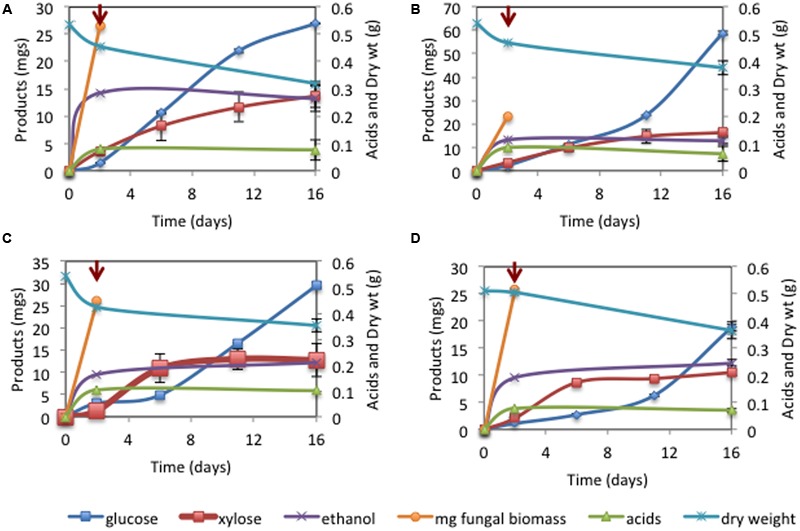
**C1A growth and metabolism of alkaline pretreated switchgrass (A)**, energy cane **(B)**, mixed prairie **(C)**, and sorghum forage **(D)**. Arrows depict time of cycloheximide addition to arrest C1A growth and initiate saccharification.

Finally, we monitored sugar extraction efficiency from four additional lignocellulosic substrates, including crop residues (sorghum forage, energy cane stems), dedicated bioenergy crops (switchgrass), as well as virgin biomass (mixed tallgrass prairie). In these experiments, total sugars, ranging between 29.23 mg (sorghum) and 75.06 mg (energy cane) were released at the conclusion of the saccharification phase, representing 6–14% of starting substrate dry weight (**Figure 5** and **Table 2**). These results demonstrate the broad applicability of this approach and the feasibility of sugar extraction from all examined lignocellulosic substrates.

**Table 2:** Sugar release from alkaline pretreated switchgrass, energy cane, sorghum, and mixed prairie by strain C1A.

Plant	Plant dry weight (g)			Products (mg)^2^						Recovery percentages^3^					
	T_0_	T_f_^1^	% lost	Acids^4^	Ethanol	Fungal biomass	Glucose	Xylose	Total ferm sugars	g sugar/ g biomass	g sugar/ g biomass	Glucose yield^5^	Xylose yield^6^	Ferment-able sugars yield^7^	Sugars: acids ratio^8^
Switchgrass	0.53	0.32	40.15	75.94 ± 13.6	13.20 ± 2.4	25.84 ± 1.59	26.98 ± 0.11	13.67 ± 2.11	40.65	0.08	0.19	10.67	7.52	9.91	0.54
Energy Cane	0.54	0.38	30.38	61.79 ± 7.9	12.77 ± 1.24	23.98 ± 1.23	58.8 ± 0.76	16.26 ± 1.41	75.06	0.14	0.32	0.54	9.20	17.19	0.86
Sorghum	0.51	0.36	28.50	70.74 ± 13.9	13.18 ± 2.67	29.8 ± 6.85	18.76 ± 0.61	10.47 ± 0.61	29.23	0.06	0.29	11.70	6.57	10.60	0.6
Mixed Prairie	0.54	0.35	35.24	100.6 ± 3.39	12.22 ± 0.59	26.14 ± 1.94	29.55 ± 0.53	12.74 ± 3.7	42.28	0.08	0.2	ND	ND	ND	0.39

*^1^Dry weight measured at the conclusion of saccharification phase. ^2^All products are measured at the conclusion of saccharification phase, with the exception of fungal biomass, which was measured at the end of the growth phase, since hyphal lysis occurred during saccharification. ^3^Recovery percentages are based on glucan, xylan, and lignin content determined for various lignocellulosic biomass utilized in this experiment and reported in Youssef et al. (2013). ^4^Values represent cumulative amounts of acetate, lactate, and formate produced. ^5^Calculated using the formula: [(g glucose produced/g glucan in starting plant)] × 100. ^6^Calculated using the formula: [(g xylose produced/g xylan in starting plant)] × 100. ^7^Calculated using the formula: [g glucose + xylose produced/g glucan and xylan in starting plant] × 100. ^8^Calculated using the formula: [g glucose + xylose produced/g acetate + formate + lactate produced] × 100.*

## Discussion

Here, we report a novel approach for the production of sugars and biofuels from lignocellulosic biomass using the anaerobic fungal isolate strain C1A and *E. coli* strain K011 in a two-stage process. The results described represent a proof of principle on the utility of anaerobic fungi for biofuel production from lignocellulosic biomass, and by no means represent an upper limit of possible yields and final products concentrations using this novel process. We anticipate yields enhancements based on improved anaerobic fungal strain selection and operational modifications (e.g., higher biomass loading, variations in pretreatment approaches) that require no scientific breakthroughs or genetic manipulations.

The use of anaerobic fungi for biofuel production from lignocellulosic biomass has multiple advantages: First, it alleviates the cost associated with using exogenous enzyme cocktails. Enzymes used for sugar extraction from lignocellulosic biomass represent a substantial part of the overall cost, estimated anywhere between $0.34 and 1.68/gallon (Humbird et al., 2011; Klein-Marcuschamer et al., 2011). Admittedly, sugar extraction efficiency (hydrolysis yield) reported here is lower than those obtained using proprietary commercial enzymes cocktails, where yields exceeding 70% for aggressively pretreated lignocellulosic substrates have frequently been reported (Van Dyk and Pletschke, 2012; Gao et al., 2014). However, it is important to note that in all microbial-based, exogenous enzymes-free saccharification approaches, a fraction of the starting plant biomass substrate will invariably be utilized for microbial growth and extracellular enzymes production. Therefore, the savings in enzymes costs counterbalance the relatively lower hydrolysis yields. Further, since C1A hyphal lysis occurs during the saccharification phase after inhibiting its growth, a fraction of plant biomass utilized for C1A growth is recovered as ethanol in the final phase of the process. Second, the proposed approach is operationally simple, with the entire process conducted in a single reaction vessel at a constant moderate temperature. The conversion of lignocellulose into desired products without added enzymes in a single reaction vessel, is regarded as the most economically viable approach for sustainable biofuel production from lignocellulosic biomass (Olson et al., 2012). Third, the proposed approach is shown to be highly effective using a greatly simplified and inexpensive treatment (alkaline pretreatment), and we reason that the localized delivery of plant biomass degradation enzymes by C1A, coupled with its physical invasiveness and disruption of plant biomass alleviate the need for complex and expensive pretreatment procedures that often leave residual chemicals or generate side products that interfere with growth of ethanologenic fermenters (Yang and Wyman, 2008; Tamrakar et al., 2011).

Exogenous enzymes-free approaches that involve the utilization of a single organism, e.g., *Clostridium thermocellum, Caldicellulosiruptor bescii* (Chung et al., 2014, 2015), *Clostridium phytofermentans* (Jin et al., 2012); or a microbial consortia, e.g., *Trichoderma reesei* and *E. coli*, have recently been an active area of research (**Table 3**). The utilization of a single organism for both saccharification and fermentation of plant biomass has been reported, although the stability of genetically modified strains, utilization of only a fraction of the substrate (e.g., C6 but not C5 sugars in *Clostridium thermocellum)*, the thermophilic nature of promising organisms (*C. thermocellum* and C. *bescii*), and the relatively low yield of the product (Chung et al., 2014) remains problematic. Our current scheme achieves higher yields than monoculture-based approaches (14.1% w/w ethanol production) by exploiting the relative strengths of two organisms (strain C1A for biomass degradation and *E. coli* strain K011 for ethanologenesis). Co-culturing efforts aim at designing microbial consortia where division of labor between a robust lignocellulolytic organism and a sugar fermenter is exploited for the production of biofuels (Minty et al., 2013; Brethauer and Studer, 2014). While scientifically fascinating, design and maintenance of stable consortia with desired proportional biomass ratios between various members is operationally challenging, and long-term predominance of a single species has often been observed. Our current approach is different from consortia-based approaches in that it employs a single living microorganism in separate, distinct phase of the process, and hence avoids problems associated with resource competition often encountered in microbial consortia.

**Table 3:** Examples of consolidated bioprocessing for biofuel production from lignocellulosic biomass.

Process type	Organism(s) used	Substrate	Product: Titer (mM)	Product yield from biomass (%)	Reference
Two stages	Anaerobic fungal strain C1A and *E. coli* strain K011	Alkaline pretreated corn stover	Ethanol: 28.16	14.1	This study
Single organism	*Caldicellulosiruptor bescii* strain JWCB033^1^	Switchgrass	Ethanol: 12.8	5.89^2^	Chung et al., 2014
	*Clostridium phytofermentans* ATCC700394	Water extracts of AFEX pretreated corn stover	Ethanol: 151.9	ND^3^	Jin et al., 2012
Co-culture	*Trichoderma reesei* RUTC30 and *Escherichia coli* strain NV3 pSA55/69^4^	AFEX pretreated corn stover	Isobutanol: 25.4	9.4^5^	Minty et al., 2013

*^1^A genetically engineered thermophilic strain of *C. bescii* DSMZ6725 constructed by deletion of lactate dehydrogenase, and the heterologous expression of a *C. thermocellum* bifunctional acetaldehyde/alcohol dehydrogenase. ^2^Calculated based on production of 12.8 mM ethanol in a 50 ml medium with 1% (0.5 g) switchgrass. ^3^ND, Not determined ^4^Genetically modified strain of *E. coli* engineered for isobutanol production (Smith and Liao, 2011); long-term predominance of a single species could occur. ^5^Calculated on the production of 1.88 g/l isobutanol from 2% AFEX pretreated corn stover.*

## Conclusion

In this work, we describe a novel approach for biofuel production from lignocellulosic biomass. The approach achieves direct, single container, exogenous enzyme-free conversion of lignocellulosic biomass to sugars and biofuels using the anaerobic fungal isolate strain C1A. This approach utilizes simple physiological manipulations for uncoupling of fungal saccharolytic and fermentative metabolism, leading to the accumulation of sugars that could subsequently be converted to biofuels. We demonstrate the feasibility of this process on mildly alkaline pretreated corn stover, as well as on a wide range of lignocellulosic biomass substrates. The potential cost savings, input and output versatility, and operational consolidation render anaerobic fungi a promising alternative for low cost biofuel production from lignocellulosic biomass.

## Author Contributions

AR, OS, CS, and NY carried out all experimental incubations and enzyme essays. HA helped in the analysis and interpretation of the results, ME conceived the study and participated in its design and coordination, AR, ME, and NY drafted the manuscript. All authors read and approved the final manuscript.

## Conflict of Interest Statement

The authors declare that the research was conducted in the absence of any commercial or financial relationships that could be construed as a potential conflict of interest.
