# Supplementary material for: Utilizing Anaerobic Fungi for Two-stage Sugar Extraction and Biofuel Production from Lignocellulosic Biomass
Source: Front Microbiol. 2017 Apr 11;8:635. doi: 10.3389/fmicb.2017.00635 (PMC5387070; doi:10.3389/fmicb.2017.00635)
Supplement: Supplementary file 1 [file Table_1.DOCX]

Supplementary document for:

Utilizing anaerobic fungi for two-stage sugar extraction and biofuel production from lignocellulosic biomass

Abhaya Ranganathan, Olivia P. Smith, Noha H. Youssef, Christopher G. Struchtemeyer, Hasan K. Atiyeh, and Mostafa S. Elshahed

**Table S1. Optimization of C1A inhibition time**

Sugar extraction from lignocellulosic biomass using C1A involves two distinct phases: a growth phase and a sugar release (saccharification) phase. It is critical to optimally allocate plant biomass substrate between both phases to ensure maximum colonization and extracellular enzyme production during the initial anaerobic phase, while maintaining a large fraction of plant biomass polysaccharides for hydrolysis and sugar accumulation during the subsequent aerobic phase. We evaluated the optimal time for arresting growth of strain C1A and initiation of the saccharification phase of the process. Strain C1A was grown for various lengths of times, ranging between 24 hours and 14 days (Column 1) before being inhibited by aeration. Saccharification phase was allowed to continue for an additional 14-day period, after which sugars (glucose and xylose) were measured in the culture supernatant (Columns 3-5). The growth time that resulted in the highest level of g sugars/g original dry weight (48 hours, bold) was deemed the optimal time for inhibition.

| **Time of inhibition** | **Corn stover dry weight at t_0_ (mg)** | **Glucose at t_f_ (mg)** | **Xylose at t_f_ (mg)** | **Total fermentable sugars at t_f_ (mgs)** | **g sugar/ g corn stover** |
| --- | --- | --- | --- | --- | --- |
| 24h | 450 | 12.43±5.48 | 18.87±8.92 | 31.30 | 0.07 |
| 36h | 450 | 28.07±6.16 | 31.62±2.05 | 59.69 | 0.13 |
| **48h** | **450** | **54.24±6.27** | **32.58±2.82** | **86.82** | **0.19** |
| 3d | 665 | 88.15±11.18 | 25.74±2.24 | 113.89 | 0.17 |
| 4d | 665 | 76.92±14.28 | 25.65±1.29 | 102.57 | 0.15 |
| 6d | 665 | 82.81±7.71 | 26.94±2.61 | 109.75 | 0.17 |
| 8d | 665 | 66.34±2.6 | 26.39±3.88 | 92.73 | 0.14 |
| 14d | 665 | 63.56±5.31 | 24.87±2.83 | 88.43 | 0.13 |

**Table S2. Effect of inhibition methods on sugar release from corn stover by strain C1A**

We compared the extent of sugar release by strain C1A from alkali-pretreated corn stover when two distinct methodologies were applied: aeration, versus the addition of cycloheximide (1mg/ml). The results demonstrate that the nature of the inhibition procedure does not impact the sugar/corn stover ratio obtained at the conclusion of the experiment.

| **Method of inhibition (at 48h)** | **Corn stover dry weight at t_0_ (mg)** | **Glucose at t_f_ (mg)** | **Xylose at t_f_ (mg)** | **Total fermentable sugars at t_f_ (mgs)** | **g sugar/ g corn stover** |
| --- | --- | --- | --- | --- | --- |
| Aeration | 483 | 57.08±2.11 | 23.87±1.66 | 80.95 | 0.17 |
| Cyclohexamide | 450 | 55.66±7.42 | 18.93±2.52 | 74.59 | 0.17 |

**Table S3. Endoglucanase, cellobiohydrolase, exoglucanase, and β-glucosidase activities in supernatant versus pellet.**

| Days post inhibition | Enzyme activities (U/mg protein; 1U=1 nmole of product produced/min for endoglucanase and cellobiohydrolase, and 1µmole of product produced/min for exoglucanase and β-glucosidase) | | | | | | | |
| --- | --- | --- | --- | --- | --- | --- | --- | --- |
|  | Endoglucanase | | Cellobiohydrolase | | Exoglucanase | | β-glucosidase | |
|  | Supernatant | Pellet | Supernatant | Pellet | Supernatant | Pellet | Supernatant | Pellet |
| 0 | 6.23±0.19 | 104.5±15.5 | 1.89±0.29 | 0.08±0.02 | 0.15±0.09 | 0.09±0.006 | 0.09±0.01 | 0.33±0.08 |
| 2 | 12.57±1.8 | 239.4±47.1 | 3.65±0.17 | 0.24±0.009 | 0.39±0.04 | 0.09±0.007 | 0.67±0.06 | 1±0.34 |
| 4 | 22.9±0.5 | 207.4±37 | 4.14±0.3 | 0.39±0.008 | 0.81±0.07 | 0.13±0.04 | 0.43±0.09 | 1.63±0.38 |
| 7 | 23.5±1.29 | 220.8±9.33 | 3.27±0.08 | 0.51±0.02 | 0.4±0.06 | 0.34±0.02 | 0.28±0.09 | 1.8±0.54 |
| 8 | 11.97±0.65 | 224.2±28.9 | 2.49±0.35 | 0.51±0.02 | 1.02±0.15 | 0.19±0.04 | 0.3±0.02 | 2.06±0.62 |
| 14 | 15.7±0.45 | 269.6±52 | 3.57±0.12 | 0.4±0.01 | 1.18±0.08 | 0.29±0.05 | 0.3±0.1 | 2.28±0.45 |

**Table S4. Xylosidase, α-N-arabinofuranosidase, xylanase, and α-glucuronidase activities in supernatant versus pellet.**

| Days post inhibition | Enzyme activities (U/mg protein; 1U=1 nmole of product produced/min for xylosidase and α-N-arabinofuranosidase, and 1µmole of product produced/min for xylanase and α-glucuronidase) | | | | | | | |
| --- | --- | --- | --- | --- | --- | --- | --- | --- |
|  | Xylosidase | | α-N-arabinofuranosidase | | Xylanase | | α-glucuronidase | |
|  | Supernatant | Pellet | Supernatant | Pellet | Supernatant | Pellet | Supernatant | Pellet |
| 0 | 0.014±0.004 | 1.1±0. 2 | ND | 2.08±0.25 | 0.38±0.04 | 0.21±0.02 | 2.51±0.33 | 1.4±0.16 |
| 2 | 0.05±0.012 | 0.89±0.07 | ND | 2.51±0.75 | 0.84±0.15 | 0.37±0.17 | 2.47±0.59 | 1.37±0.46 |
| 4 | 0.043±0.03 | 0.3±0.03 | ND | 2.53±0.05 | 1.23±0.15 | 0.71±0.12 | 3.61±0.2 | 1.74±0.33 |
| 7 | 0.043±0.02 | 0.32±0.02 | ND | 3.8±0.09 | 0.74±0.25 | 1.03±0.32 | 1.57±0.08 | 1.15±0.12 |
| 8 | 0.019±0.001 | 1.24±0.02 | ND | 4.11±0.01 | 0.65±0.12 | 0.83±0.03 | 3.19±0.41 | 1.48±0.16 |
| 14 | 0.022±0.001 | 3.16±0.09 | ND | 5.15±0.09 | 0.85±0.22 | 0.92±0.31 | 3.23±0.85 | 0.14±0.07 |
